# Cardiac manifestations in adult patients with inherited metabolic disease: A single-center experience

**DOI:** 10.1016/j.ymgmr.2025.101216

**Published:** 2025-04-11

**Authors:** Flutura Sadiku, Tobias Rutz, Andrea Superti-Furga, Pierre Monney, Christel Tran

**Affiliations:** a Division of Genetic Medicine, University of Lausanne and University Hospital of Lausanne, Lausanne, Switzerland; b Service of Cardiology, Lausanne University Hospital and University of Lausanne, Lausanne, Switzerland; c Genetica AG, Zurich, Switzerland

**Keywords:** Inherited metabolic disease, Cardiac disease, Structural heart defect, Valvular disease, Arrythmia

## Abstract

**Background:**

Inherited metabolic diseases (IMDs) can affect the heart, but data on cardiac manifestations in adults are scarce. This study examines the clinical and radiological features of cardiac complications in adults with IMDs.

**Methods:**

This retrospective study included adult patients at our metabolic clinic with a biochemical and/or genetic diagnosis of IMD who underwent cardiac investigations. Records were reviewed for clinical features, echocardiograms, electrocardiograms, and treatment. Patients were categorized into three IMD subgroups: disorders of small molecules, complex molecules, and energy production.

**Results:**

Of the 115 adult patients with IMD, 48 underwent cardiac testing (mean age 39.1 ± 14.8 years). Abnormal cardiac findings were reported in 23 of these patients (47.9 %, 14 men). Five (21.7 %) were symptomatic with dyspnea, peripheral edema, or chest pain. Fourteen patients (60.9 %) had heart muscle disease, 6 (26.1 %) had valvular involvement, and 5 (21.7 %) had arrhythmia. Valvular and heart muscle disease predominated in complex and small molecule disorders (3/4 and 7/9 respectively). Energy production disorders showed mixed involvement: heart muscle disease (5/10) and arrhythmia (5/10). Twelve of the 23 patients with abnormal findings (52.2 %) received specific cardiac therapy. All but one patient remained stable under treatment.

**Discussion:**

In this cohort, cardiac disease was diagnosed in 23 of 115 adults with IMD (20 %), including structural heart defects and arrhythmia. The pattern and severity of cardiac involvement varied between disorders, with arrhythmia mainly associated with energy production disorders. Outcomes were favorable in most cases, likely due to collaboration between metabolic physicians and cardiologists and timely follow-up and treatment.

## Highlights

• Inherited metabolic diseases (IMD) result from genetic enzyme or transport protein variants.
• IMDs affect metabolism and can lead to systemic or organ-specific issues, including the heart.
• Cardiac dysfunction, such as heart failure or arrhythmias, is potentially a significant IMD complication.
• Studies on IMD and cardiac disease mainly focus on pediatric cases, not adults.
• This study examined cardiac function in 48 adult IMD patients, finding 47.9 % with abnormalities.

## 1 Introduction

Inherited metabolic diseases (IMD) are the consequence of genetic variation in enzymes or transport proteins that affect the metabolism of protein, fat, or carbohydrate, or impair organelle function. Over 1400 conditions have been classified as IMD (or inborn errors of metabolism) [1]. As a group, IMDs have a birth prevalence estimate of approx. 50.9 per 100,000 live birth, with some differences depending on the region of the world studied [2,3]. Improvements in screening programs, disease awareness, diagnostic tests and therapeutic interventions in IMD have led to longer patient survival and better prognosis, but also to the detection of milder variants or late-onset forms that present in adulthood [[4], [5], [6]].

Clinical manifestation of IMD can be acute or progressive and can involve a specific organ or be systemic. As IMD can affect the primary sources of energy (glycogen or fatty acids), they can lead to cardiac dysfunction. Although most of IMDs are multisystemic, cardiac dysfunction may be the main clinical feature and source of major complications, such as heart failure or arrhythmias. More than 40 types of IMDs have been associated with cardiac dysfunction [[7], [8], [9], [10]], albeit with different patterns and variable severity. Most studies and reports on IMD and cardiac disease concern the pediatric population [[11], [12], [13], [14]]. In the pediatric age range, 5 to 30 % of cardiomyopathies are caused by IMD; these include mainly fatty acid oxidation disorders, glycogen storage disorders, lysosomal storage disorders, mitochondrial disorders and organic acidemias [15,16]. The CardioMetabo study done on a cohort of 59 adult patients ascertained by hypertrophic cardiomyopathy (HCM) identified 6 patients with IMD [17].

While there is no doubt that recognizing cardiac involvement in pediatric patients with IMD can lead to better outcomes with disease-specific treatments, little data is available regarding cardiac disease in adult IMD patients. The aim of this study was to obtain information on the prevalence and the features of cardiac involvement and its clinical outcome in a cohort of adult patients with IMD followed in a dedicated clinic within a reference center for rare diseases (Lausanne University Hospital, CHUV).

## 2 Material and methods

We included all patients older than age 16 years seen at our Clinic between 2013 and 2020 who had an unequivocal biochemical and/or genetic diagnosis of IMD and who had cardiologic studies including at least a clinical examination and a cardiac ultrasound. Cardiac investigations were ordered on an individual basis on the indication of either [1] clinical signs or symptoms such as dyspnea, edema, palpitations, chest pain or [2] a diagnosis of an IMD known to be associated with cardiac involvement. The primary endpoint was the confirmation (or exclusion) of cardiac abnormalities such as electrocardiogram (ECG) abnormalities, valvular involvement, or ventricular abnormalities (hypertrophy, dilatation, fibrosis, remodeling). Secondary endpoints included symptoms related to cardiac abnormality, associated cardiovascular risk factors and therapy modalities.

Patients were divided into three groups according to the pathophysiology and using a simplified classification of IMD: group 1; *complex molecules disorders,* group 2; *small molecules (intoxication) disorders* and group 3; *energy defect disorders* [18]. Electronic and paper patient records from the Divisions of Genetic Medicine and of Cardiology were reviewed for clinical features including age, sex, anthropometric measurements, age at the time of analysis, at diagnosis of IMD and at detection of cardiac abnormality, treatment (drug medications and other interventions) as well as for the results of cardiologic studies such as transthoracic echocardiographic (TTE) findings, electrocardiogram (ECG), and Holter recordings. Treatment included specific therapies for IMD (drugs, vitamins, enzymatic cofactors, amino acid supplements, enzyme replacement therapy, liver transplant) as well as for cardiac disease (drugs, thermo-ablation, pacemaker, stenting procedures).

Comprehensive TTE was performed according to the recommendations for cardiac chambers quantification of the American Society of Echocardiography and the European Association of Cardiovascular Imaging [19]. Disorders of the heart muscle included hypertrophic cardiomyopathy (HCM), left ventricular hypertrophy (LVH), hypokinetic left ventricle, left and/or right systolic dysfunction, left ventricular (LV) concentric remodeling, left or right ventricular dilatation, dilated cardiomyopathy (DCM) and non-compaction cardiomyopathy.

LV was considered dilated in case of an increased LV diastolic volume index (>75 ml/m^2^ in men or > 61 ml/m^2^ in women); if no LV volumes were available, dilatation was defined as an increased LV diastolic diameter (>58 mm in men or > 52 mm in women). LV hypertrophy (LVH) was defined as an increase in LV mass index above normal limits (>115 g/m^2^ in men or > 95 g/m^2^ in women) while concentric remodeling was defined as an increase in relative wall thickness (>0.42) in the presence of a normal LV mass index. RV dilatation was defined as an increase in RV basal diameter or RV diastolic area on an apical 4-chamber view and RV dysfunction was defined as a decrease in TAPSE (tricuspid annular plan systolic excursion), a decrease in systolic annular plane velocity or a decrease in RV fractional area change below the reference range. Dilated cardiomyopathy (DCM) was defined as a dilated LV with systolic dysfunction, which cannot be explained by abnormal loading conditions (e.g. valvular regurgitation). Hypokinetic LV was defined as a LV with reduced EF but no dilatation. Hypertrophic cardiomyopathy (HCM) was defined as a localized thickening of the LV wall (≥15 mm) with or without increase in LV mass index, which cannot be explained by abnormal loading conditions (e.g. hypertension, aortic stenosis) [20]. LV isolated non-compaction was defined according to the Jenni criteria by echocardiography [21] or the Petersen criteria by cardiac magnetic resonance [22]. Valvular abnormalities were considered secondary to the metabolic disease only in case of alteration of leaflets or cusps morphology, such as sclerosis, retraction or thickening. Functional valve regurgitations (consecutive to aortic root dilatation or ventricular dilatation/dysfunction) were not considered as directly caused by the metabolic disease [23]. Pulmonary hypertension was defined according to the European Society of Cardiology (ESC) and European Respiratory Society (ERS) guidelines [24].

Patients with Fabry disease (OMIM# 301500) were not included in this study as they are followed by a separate specific clinic at our Institution and their data have been published previously [25].

All data were entered in the database (excel file) and were analyzed by research team members (FS, CT, PM and TR). The protocol was approved by the Swiss Ethics Committees on research involving humans (Approval # 2020–02531) and was registered at the U.S Clinical Trials Registry as NCT04999566.

### 2.1 Statistical analysis

Data were analyzed using Excel statistical functions for Office 365 for Mac and for Windows 2016. Frequency was calculated for classification of patients according to the metabolic subtype. Prevalence was reported as percentage of the total group or subgroup of patients depending on the studied outcome. Data for age at the time of analysis were presented as mean ± SD.

## 3 Results

### 3.1 General results

Of the 115 patients aged 16 and older followed in our IMD clinic, 48 patients (42 %; 25 men and 23 women) had had cardiac investigations and were included in the study. Patients with IMD are monitored at regular intervals in our metabolic clinic, with the frequency adjusted based on the type of disorder, the involvement of target organs, and the treatments in place. Among the 48 patients who had cardiac investigations, 22 (45.8 %), 24 (50 %) and 2 (4.2 %) were followed 1×/yrs., 2×/yrs. and > than 2×/yrs., respectively.

Among the 67 (58.3 %) patients without documented cardiac evaluation, the majority had IMD that are not typically associated with an increased risk of cardiac disease and were asymptomatic. These primarily included small molecule disorders (i.e. phenylketonuria, urea cycle disorders, biotinidase deficiency, classical galactosemia). Other factors contributed to the exclusion of a subset of patients from the study. Specifically, four patients did not consent to data sharing, one declined cardiac screening, one passed away before consent could be obtained, and four were lost to follow-up or transferred to another clinic before cardiac investigations could be performed.

**Fig. 1:**
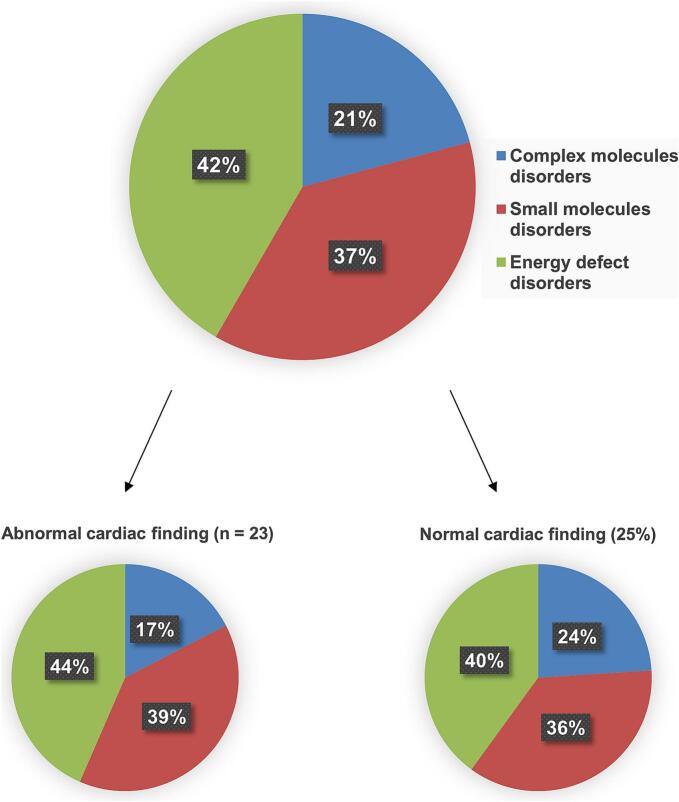
Repartition of patients with inherited metabolic diseases who had cardiac studies in this cohort (*n* = 48).

Among the 48 patients who had had cardiac investigations, 23 (47.9 %) had cardiac abnormalities (14 men, 9 women) whereas the remaining 25 patients (52.1 %) had no cardiac abnormality related to IMD (Additional file 1). An overview of the main results in patients with cardiac abnormalities are summarized in Table 1 and Table 2. The proportion of patients who had cardiac abnormalities varied depending on the diagnostic subgroup. Thus, 4/23 of the large molecules group had cardiac abnormalities (17.4 %), as compared to 9/23 in the small molecules (39.1 %) and 10/23 (43.5 %) in the energy group (Fig. 1). In most patients (17/23, 73.9 %), cardiac abnormalities were first reported in adulthood, likely several years after the IMD diagnosis (mean 10.1 ± 4.5 years), based on available reports.

**Table 1:** Cardiac findings and age at detection of cardiac abnormality.

ID	IMD	#OMIM	Current age (y), sex	Age (y) at diagnosis of IMD	Cardiac findings	Age (y) at detection of cardiac abnormality
**Group 1: COMPLEX MOLECULES DISORDERS**						
1	MPS IVA (Morquio)	253000	29, F	4	Aortic and mitral valve leaflet thickening	∼12
2	MPS II (Hunter)	309900	33, M	6	Severe aortic insufficiency with LV dilatation	11
3	NPD-B	607616	30, F	Birth	Intermediate probability of PH	29
4	NPD-B	607616	58, M	51	LV dilatation, mild aortic valve sclerosis, intermediate probability of PH	59
**Group 2: SMALL MOLECULES DISORDERS**						
5	Argininosuccinic aciduria	207900	37, F	1	Reversible DCM	33
6	PA	606054	39, M	2	DCM, LV dilatation, LVH	18
7	Cobalamin C deficiency	277400	33, M	1 mo	Hypokinetic LV	26
8	Cobalamin C deficiency	277400	23, M	Birth	LV non-compaction CM	16
9	Cobalamin C deficiency	277400	21, M	Birth	LV non-compaction CM Reversible DCM Mild RVSD	Birth
10	Classical homocystinuria	236200	28, M	5	Aortic root dilatation, mild aortic valve insufficiency	26
11	Classical homocystinuria	236200	48, F	38	DCM, mild LVSD	44
12	Classical homocystinuria	236200	48, M	43	Aortic root dilatation	43
13	Classical homocystinuria	236200	57, F	51	LV concentric remodeling Aortic root dilatation	51
**Group 3: ENERGY DEFECT DISORDERS**						
14	GSD IIIa	232400	22, F	6	HCM, prolonged QTc	10
15	GSD IIIa	232400	42, M	2	LV concentric remodeling	NA
16	Leigh syndrome	256000	74, F	63	Aortic sclerosis with mild regurgitation	68
17	Maternally-inherited CPEO	157640	32, M	26	Aspecific intramyocardial fibrosis	34
18	MELAS	540000	63, M	48	LV concentric remodeling Mild left atrial dilatation, Coronary heart disease	59
19	MELAS	540000	died aged 31, M	19	LVSD, hypokinetic LV, RV dilatation Severe functional tricuspid insufficiency WPW syndrome	18
20	KSS	530000	31, F	18	Right bundle branch block, left anterior fascicular block	22
21	KSS	530000	68, M	51	High degree AV block Left atrial dilatation Coronary heart disease	56
22	X-linked CRTR-D	300352	32, F	24	Repolarization abnormalities	32
23	CTD	212140	40, M	6	Reversible DCM	6

CM; cardiomyopathy, CPEO; chronic progressive external ophthalmoplegia, CTD; carnitine transporter deficiency, CRTR-D; creatine transporter defect, DCM; dilated cardiomyopathy, EF; ejection fraction, GSD IIIa; glycogen storage disease type IIIa, HCM; hypertrophic cardiomyopathy, LVSD; left ventricular systolic dysfunction, KSS; Kearns-Sayre syndrome, LV; left ventricle, LVH; left ventricular hypertrophy, LVSD; left ventricular systolic dysfunction, MELAS; mitochondrial encephalopathy with lactic acidosis and stroke-like episodes, MPS; mucopolysaccharidosis, NPB; Niemann Pick disease, PA; propionic academia, PH ; pulmonary hypertension, RV; right ventricular, RVSD: right ventricular systolic dysfunction, WPW; Wolf Parkinson White syndrome.

NA: pediatric data unavailable.

**Table 2:** Symptoms, cardiovascular risk factors and/or events and treatments.

ID	IMD	#OMIM	Cardiac symptoms	CV risk factors/events	Cardiac treatments	Other treatments⁎
**Group 1: COMPLEX MOLECULES DISORDERS**						
1	MPS IVA (Morquio)	253000	Dyspnea	–	–	Idursulfase (ERT)
2	MPS II (Hunter)	309900	Dyspnea grade II	–	ACE inhibitor	Alglucosidase alpha (ERT)
3	NPD-B	607616	Dyspnea grade II, palpitations, chest pain	Dyslipidemia, smoking	–	Calcium, vitamin D3
4	NPD-B	607616	Dyspnea grade IV, legs edema	Hyperlipidemia,	Beta blocker	Calcium, vitamin D
**Group 2: SMALL MOLECULES DISORDERS**						
5	Argininosuccinic aciduria	207900	No symptom	–	–	L-arginine, vitamin B12 and B9
6	PA	606054	No symptom	Stroke, HTA	ACE inhibitor, diuretic, beta blocker	l-carnitine, liver transplant
7	Cobalamin C deficiency	277400	No symptom	Dyslipidemia, HTA	ACE inhibitor	Betaine, vitamin B12 and B9, L-methionine, l-carnitine
8	Cobalamin C deficiency	277400	No symptom	Overweight		Betaine, l-carnitine, vitamin B12, B9, L-methionine
9	Cobalamin C deficiency	277400	No symptom	Overweight	ACE inhibitor,	Betaine, vitamin B6, B12,B9
10	Classical homocystinuria	236200	No symptom	–	–	Vitamin B6, B12, B9, betaine, amino acids supplements
11	Classical homocystinuria	236200	No symptom	–	–	Vitamin B6, B12, B9, anticoagulant
12	Classical homocystinuria	236200	No symptom	Dyslipidemia, HTA	ACE inhibitor, diuretic, beta blocker	Vitamin B6, B12, B9
13	Classical homocystinuria	236200	No symptom	Obesity, hypercholesterolemia, HTA	AT2 antagonist	Vitamin B6, B12, B9
**Group 3: ENERGY DEFECT DISORDERS**						
14	GSD IIIa	232400	No symptom	–	Beta blocker,	Liver transplant
15	GSD IIIa	232400	No symptom	Dyslipidemia, smoking	–	
16	Leigh syndrome	256000	No symptom	Diabetes, HTA	ACE inhibitor, diuretic, Ca antagonist	Co-enzyme Q10, vitamin C, complex B vitamins
17	Maternally-inherited CPEO	157640	No symptom	–	–	Co-enzyme Q10, creatine
18	MELAS	540000	Chest pain	Dyslipidemia, overweight, HTA	ACE inhibitor, diuretic, statin, beta blocker	
19	MELAS	540000	NA	Type 2 diabetes	ACE inhibitor, diuretic, thermoablation of 3 accessory bundles	Co-enzyme Q10, L-arginine, L-cartinine,
20	KSS	530000	No symptom	–	–	Co-enzyme Q10, creatine
21	KSS	530000	No symptom	Diabetes, obesity, hypercholesterolemia, family history	Pacemaker, stent, ACE inhibitor, beta blocker, statin	–
22	X-linked CRTR-D	300352	No symptom	–	–	L-arginine, L-glycine, creatine
23	CTD	212140	No symptom	HTA	–	l-carnitine, B complex vitamins

ACE; angiotensin-converting enzyme, AT2; angiotensin 2 receptor antagonist, CPEO; chronic progressive external ophthalmoplegia, CTD; carnitine transporter deficiency, CRTR-D; creatine transporter defect, DCM; dilated cardiomyopathy, EF; ejection fraction, ERT; enzyme replacement therapy, GSD IIIa; glycogen storage disease type IIIa, HCM; hypertrophic cardiomyopathy, HTA; hypertension, KSS; Kearns-Sayre syndrome, LV; left ventricle, LVH; left ventricular hypertrophy, MELAS; mitochondrial encephalopathy with lactic acidosis and stroke-like episodes, MPS; mucopolysaccharidosis, NPB; Niemann Pick disease, PA; propionic academia, RV; right ventricle.

⁎ Specific treatments related to IMD including drugs, vitamins, cofactors, amino acid mixtures, liver transplant.

Of the 48 patients who had cardiac investigations, the mean age (in years) at the time of our chart review was 40.26 (± 12.9), 35.5 (± 13.6) and 41.5 (±16.8), respectively, in the three subgroups (complex molecules, small molecules and energy defects). Among the patients with cardiac disease, the median age at detection of cardiac anomalies was 20.5 years (age ranged from 11 to 52 years) for complex molecules disorders, 26 years (0 to 51 years) for small molecules disorders, and 32 years (6 to 68 years) for energy defect disorders.

### 3.2 Indications for cardiac studies

Only five patients (21.7 %) presented with symptoms related to the cardiac involvement and/or pulmonary involvement including dyspnea, leg edema, palpitations, and chest pain. For one patient with Cobalamin C deficiency (ID 9), cardiac involvement was one of the presenting signs of the IMD, along with other systemic manifestations. All the other patients in this cohort were investigated in the context of follow-up cardiac investigations.

### 3.3 Cardiac studies

All the included patients had a transthoracic echocardiogram (TTE). TTE findings were reported in Table 3. Sixteen patients (69.6 %) had an ECG, 6 patients (26.1 %) a Holter monitor, 3 patients (13 %) a cardiac stress test, 8 patients (34.8 %) a cardiac magnetic resonance imaging (with available data) and one patient had a Schellong orthostatic hypotension test.

**Table 3:** Transthoracic echocardiogram findings.

ID	IMD	Age, Sex	LV diastolicdiameter (mm)	LV diastolicvolume index (ml/m^2^)	LV ejection fraction (%)	Maximal wall thickness (mm)	Relative wall thickness	LV mass index (g/m^2^)	LV Morphology	RV Morphology	RV function	Probability of PH	Aortic root *Z*-score
1	MPS IVA	29, F	34	37	72	5	0.24	34	Normal	N	N	Low	1.2
2	MPS II	33, M	58	93 (↑)	60	6	0.21	102	Dilatation	N	N	Low	1.5
3	NPD-B	30, F	49	55	64	9	0.37	86	Normal	N	N	Intermediate	0.6
4	NPD-B	58, M	59 (↑)	83 (↑)	55	7	0.24	91	Dilatation	N	N	Intermediate	0.4
5	Arginino-succinc aciduria	37, F	51	53	59	8	0.31	66	Normal	N	N	Low	−1.2
6	PA	39, M	64 (↑)	112 (↑)	46 (↓)	10	0.28	123 (↑)	Dilatation, LVH	N	N	Low	−2.0
7	Cobalamin C deficiency	33, M	51	48	47 (↓)	12	0.39	103	Hypokinetic	N	N	Low	0.5
8	Cobalamin C deficiency	23, M	55	40	55	10	0.29	99	Non-compaction	N	N	Low	−2.0
9	Cobalamin C deficiency	21, M	54	57	56	10	0.37	94	Non-compaction	N	RV dysfunction	Low	0.8
10	Classical homocystinuria	28, M	54	70	58	10	0.26	80	Normal	N	N	Low	2.1
11	Classical homocystinuria	48, F	51	56	54	9	0.35	80	Normal	N	N	Low	0.6
**ID**	**IMD**	**Age, Sex**	**LV diameter (mm)**	**LV volume index (ml/m**^**2**^**)**	**LV ejection fraction (%)**	**Maximal wall thickness (mm)**	**Relative wall thickness**	**LV mass index (g/m**^**2**^**)**	**LV Morphology**	**RV Morphology**	**RV function**	**Probability of PH**	**Aortic root** **Z-score**
12	Classical homocystinuria	48, M	51	53	54	10	0.35	87	Normal	N	N	Low	2.7
13	Classical homocystinuria	57, F	51	Normal*	Normal*	11	0.43 (↑)	112	Concentric remodeling	N	N	Low	3.2
14	GSD IIIa	22, F	47	48	72	18 (↑)	0.30	119 (↑)	HCM	N	N	Low	0.1
15	GSD IIIa	42, M	40	38	70	12	0.55 (↑)	89	Concentric remodeling	N	N	Low	−1.1
16	Leigh syndrome	74, F	42	Normal*	65	8	0.33	62	Normal	N	N	Low	−1.5
17	Maternally-inherited CPEO	32, M	53	70	60	8	0.30	76	Normal	N	N	Low	0
18	MELAS	63, M	38	52	58	10	0.42 (↑)	70	Concentric remodeling	N	N	Low	−0.9
19	MELAS	died at 31, M	47	48	22 (↓)	9	0.38	93	Hypokinetic	Dilated	RV dysfunction	N/A	−2.3
20	KSS	31, F	42	29	54	6	0.29	53	Normal	N	N	Low	−1.5
21	KSS	68, M	59 (↑)	Normal*	65	11	0.37	101	Normal	N	N	Low	−1.8
22	X-linked CRTR-D	32, F	45	53	68	6	0.27	44	Normal	N	N	Low	−0.4
23	CTD	40, M	56	74	53	9	0.35	92	Normal	N	N	Low	0.9

GSD IIIa; glycogen storage disease type IIIa, CPEO; chronic progressive external ophthalmoplegia, CRTR-D; creatine transporter defect, CTD; carnitine transporter deficiency, HCM; hypertrophic cardiomyopathy, KSS; Kearns-Sayre syndrome, LV; left ventricle, LVH; left ventricle hypertrophy, MELAS; mitochondrial encephalopathy with lactic acidosis and stroke-like episodes, MPS; mucopolysaccharidosis, NPB; Niemann Pick disease, PA; propionic academia, PH; pulmonary hypertension, RV; right ventricle. *Quantitative value not available.

### 3.4 Therapeutic approaches

Twelve patients (52.2 %) were given a specific therapy for their heart condition (drugs, surgery or assist devices). Most of these patients (8/12) were also treated with specific therapies for IMD. All except one patient (ID 19; MELAS) had a stable cardiac function under treatment. ID 6 (propionic acidemia) developed LVH and DCM at the age of 18 and was treated with ACE inhibitor, diuretic and beta blocker (Table 2). He had liver transplant at age 22 years. His cardiac function remained stable since then.

### 3.5 Specific cardiac manifestations

#### 3.5.1 Disorders of the heart muscle

Disorders of the heart muscle was the most prevalent cardiac manifestation. It affected 14 patients (60.9 %, Table 1). Patients from the three groups of disorders presented with heart muscle involvement. Heart muscle disorders were predominant in small molecules disorders 77.8 %, 7/9) and energy defect disorders (50 %, 5/10). ID 19 (MELAS), presented with LVH and coronary heart disease 9 years and 13 years respectively, after the diagnosis of IMD.

Four patients with small molecules disorders had DCM: arginosuccinic aciduria (ID 5), propionic acidemia (ID 6), cobalamin C deficiency (ID 9), classical homocystinuria (ID 11) and 1 patient in energy defect disorder (carnitine transporter deficiency, ID 23). ID 5 (arginosuccinic aciduria) developed transitory DCM secondary to anemia, which then resolved with normalization of blood tests. Congenital DCM was detected at birth in ID 9 (cobalamin C deficiency). DCM resolved with metabolic and cardiac treatment, although non-compaction cardiomyopathy persisted into adulthood. ID 23 (carnitine transport deficiency) had DCM during infancy, which then resolved under carnitine supplementation.

#### 3.5.2 Primary valvular involvement

Six patients (26.1 %) presented with primary valvular involvement. Three patients with complex molecules disorders (mucopolysaccharidosis (MPS): ID 1 & 2 and NPD-B: ID 4), two patients with mitochondrial disorders (ID 16, and ID 19) and one patient with classical homocystinuria (ID 10).

ID 1 (MPS type IVA) had aortic and mitral valve leaflet thickening and ID 2 (MPSII) had severe aortic insufficiency (Table 1). Due to his multiple comorbidities, it was decided not to repair the aortic valve surgically in ID 2 but to treat conservatively with an angiotensin-converting enzyme inhibitor. ID 4 (NPD-B) had mitral and aortic sclerosis. (Table 1). ID 10 (classical homocystinuria) presented with mild aortic valve insufficiency but normal EF (58 %). ID 16 (Leigh syndrome) had aortic sclerosis with mild regurgitation which could also be related to aging and still considered as physiologic (Table 1). ID 19 (MELAS) suffered from severe biventricular systolic and diastolic dysfunction, RV dilatation and severe functional tricuspid insufficiency.

#### 3.5.3 ECG abnormalities

ECG abnormalities were detected in 50 % (5/10) of the patients with energy defect disorders, including glycogen storage disease (ID 14), mitochondrial disorders (ID 19, 20 and 21) and X-linked creatine transport defect (ID 22). Five patients (21.7 %) had ECG abnormalities.

ID 14 (GSD type III), treated with liver transplantation at the age of 13, had HCM and a prolonged QTc during childhood. This subject with GSD III carried a pathogenic variant in *ALPK3*, which may have contributed, alongside GSD III, to the development of HCM and prolonged QTc [26]. ID 19 (MELAS) mentioned above for severe heart failure and functional tricuspid regurgitation, also had Wolf Parkinson White syndrome for which he was treated with thermo-ablation of three accessory bundles. He died at the age of 31 from natural history of MELAS and progressive cardiac insufficiency with a low ejection fraction (EF) of 15 % at his last evaluation. Two patients with Kearns-Sayre syndrome (KSS) presented with heart block (ID 20 and ID 21) reported at the age of 22 and 56, respectively. Repolarization abnormalities were found in one patient with X-linked creatine transporter defect (ID 22). None of these three patients were symptomatic and TTE exams were otherwise normal (Table 3).

#### 3.5.4 Aortic dilatation

Aortic abnormalities were reported in three patients (13 %) with classical homocystinuria (ID 10, 12 and 13). All of them had dilatation of the root and/or of the ascending aorta. ID 13 had concomitant LV concentric remodeling and was treated with an angiotensin II receptor antagonist (Additional file 1).

#### 3.5.5 Cardiovascular disease risk factors

Cardiovascular disease risk factors including arterial hypertension, smoking, dyslipidemia, diabetes mellitus, overweight or obesity were observed in 14 patients with cardiac involvement (61 %) and in 12 patients in the group without cardiac abnormalities (52 %, Table 2). The risk factors were distributed between the three groups without predominance for one of the groups or types of diseases. Coronary heart disease was reported in two patients with mitochondrial disorders (ID 18, MELAS and ID 21, KSS) who individually cumulated several risk factors (diabetes, dyslipidemia, obesity, Table 2). ID 29 (GSD II, no cardiac abnormality related to IMD) presented with ischemic cardiopathy likely related to heavy smoking (Additional file 1).

## 4 Discussion

Most inherited metabolic disorders are pleiotropic and can lead to multisystem disease. Nevertheless, knowledge on cardiac involvement in adult individuals with IMDs is limited to sporadic case reports or small series. To date, there is no global recommendation for screening for cardiac involvement in patients with IMD. Thus, cardiac evaluations are done on a case-by-case basis either based on clinical signs and symptoms, or because of current literature reports of cardiac involvement in specific conditions.

This retrospective chart review study was conducted to obtain a first broader insight on the incidence and the features of cardiac involvement in adults with IMD. The finding of cardiac disease in overall 20 % (23/115) of adult IMD patients is significant. In addition, this figure is likely an underestimation, as only 48 of 115 patients in our cohort had undergone cardiac evaluation. In line with observations of pediatric cases, the type and severity of cardiac involvement varies according to the disease group and its pathophysiology.

### 4.1 The pattern of cardiac injury in this series differed between disease groups

Cardiac involvement in the ***complex molecules disorders*** are mostly linked to the accumulation of partially degraded macromolecules within lysosomes as well as in the extracellular matrix and involves myocardium, heart valves, conduction tissue and vascular endothelium. It can develop early or late in life depending on the rapidity of progression in the different forms of MPS [27]. Valvular involvement is a typical feature of some MPS and was the most prevalent cardiac defect in our cohort of patients with complex molecules disorders. ID1 and 2 (MPS type IVA and type II) were diagnosed with valvular aortic and mitral involvement during infancy, which remained stable in adulthood with conservative treatment. The mechanism is partly due to accumulation of glycosaminoglycans in valve tissue, mainly in the mitral valve followed by aortic valve, leading to stenosis and regurgitation [28,29].

In ***small molecules disorders***, the disease pathogenesis is believed to be linked, in general, to the accumulation of abnormal and potentially toxic intermediary products caused by the metabolic block [11]. In our study, heart muscle disorders were the most prevalent cardiac manifestations in this group. It was identified in patients with Cobalamin C deficiency (cblC), classical homocystinuria and propionic acidemia. Non compaction cardiomyopathy was observed in two patients with cblC (ID 8 and 9). The fact that number of other cblC patients have been reported to have non compaction cardiomyopathy supports the hypothesis of a specific association between cblC and cardiac pathology; defective intracellular cobalamin metabolism might impair myocyte differentiation and myocardial maturation [30].

As the heart is highly dependent on oxidative energy, patients with ***energy defect disorders*** may be particularly at risk to develop cardiac manifestations. Indeed, CM, atrial and ventricular arrhythmias as well as conduction defects have been reported in association with mitochondrial disorders in adults and/or children [[31], [32], [33], [34], [35], [36], [37]]. Of our 20 patients with energy defect disorders, 10 had cardiac manifestations. In most of them, conduction abnormalities developed in adulthood, several years after their IMD had been diagnosed.

### 4.2 Adult patients may have milder and silent forms of cardiac involvement and/or long-term complications diagnosed later in the disease course

One patient with Niemann Pick disease type B (ID 4) presented with mild valvular involvement. Valvular involvement occurs in a minority of adult or pediatric patients with NPD and may contribute to morbidity [[38], [39], [40]]. In a cohort of 103 NPD-B adult and pediatric patients, 5 patients presented with valvular involvement [41]. Three of them had valvuloplasty and one of them died decades later of heart failure. The mechanism of valve involvement is yet unknown, but histology examinations have shown lipid deposits in histiocytes and increased number of these cells in the myocardium, valves, and pericardium [42].

Patients with classical homocystinuria in the present cohort had valve disease, aortic dilatation and/or DCM but no cardiac symptoms. Little is known about the occurrence of heart muscle disorders in classical homocystinuria [43]. Cardiac involvement is likely due to vascular and connective tissue fragility [44]. Chronically elevated homocysteine, by interfering with fibrillin-1 disulfide bonds, may lead to changes in the cardiac structure including aortic dilation and valvulopathies [45,46]. Arterial hypertension, which is estimated to occur in 30 % of cases of classical homocystinuria [43,46] may contribute to cardiomyopathy in adults. Nevertheless, the relationship between arterial hypertension and classical homocystinuria remains controversial and the contribution of aging and other comorbidities remains to be clarified.

Cardiac involvement may be silent and thus escape detection. Asymptomatic LVH is a typical finding in GSD III. ID 15 (GSD IIIa) presented with asymptomatic concentric remodeling of the left ventricle. Although most patients remain free from cardiac symptoms, arrhythmias, heart failure, prolonged QTc and even major events like sudden death have been reported in GSD III [47,48]. In the subgroup of GSDs with heart involvement, periodic echocardiography and ECG may be recommended [49,50].

### 4.3 Appropriate and timely specific treatment may reverse the cardiac manifestation or stabilize the condition of the patient

Congenital cardiomyopathy has repeatedly been reported in children with cobalamin C disease [30,51,52]. Severe heart failure with low EF (20 %) occurred at birth in patient ID 9 and prompted a metabolic work-up, leading to the diagnosis of Cobalamin C with dilated CM. Treatment of heart failure and of the underlying metabolic decompensation led to an improvement in EF and ultimately to partial recovery of CM.

ID 6 (propionic acidemia, PA) was diagnosed with DCM and LVH at the age of 18 and underwent liver transplantation at 22. Both DCM and HCM are well-documented complications of PA and can, in some cases, be the initial manifestation of the disease, even before metabolic decompensation. Liver transplantation may improve or stabilize CM in PA, likely by reducing the accumulation of toxic metabolites in the blood and tissues [53]. However, additional pathophysiological mechanisms may be involved, as good metabolic control alone does not always prevent the development of DCM. These observations highlight the importance of regular cardiac follow-up in PA, as cardiomyopathy can emerge at any age and may be stabilized—or potentially prevented—with liver transplantation [54]. In cases of unexplained cardiomyopathy, an underlying IMD should be considered, as some forms may benefit from specific treatments.

Therapeutic approach to cardiac manifestations in IMD is similar to that used in common cardiac diseases; thus, heart failure is treated with angiotensin-converting enzyme (ACE) inhibitors, β-blockers, and diuretics [55]. Approx. half of the IMD patients (52.2 %) with cardiac involvement received specific cardiac treatment, while the remaining patients were followed up regularly by cardiologists. All of them, except for one patient who died of MELAS syndrome (ID 19), remained stable. In MPS, enzyme replacement therapy is effective for cardiomyopathy but do not seem to prevent valve disease [56]. The avascular nature of cardiac valve tissue and the accumulation of glycosaminoglycans leading to retraction of the leaflets may, partly, explain the lack of benefit [57,58]. ID 2 (Hunter disease) was treated with ERT for 13 years. His severe aortic insufficiency and ventricular dilatation remained stable over the past 10 years under ACE inhibitor treatment. Of note, the ACE inhibitor was introduced to stabilize the ventricular dilatation and function as the patient is deemed to be inoperable due to his severe comorbidities, although such a treatment has not been shown to prevent the need of valve replacement in the general population with aortic regurgitation.

### 4.4 Limitations of the study

This study has several important limitations. Despite the primary cohort including 115 adult patients followed in our clinic, only 48 had a cardiac study and it remains uncertain whether those who did not have a cardiac study had normal heart function and structure or not. Secondly the underlying diagnoses are heterogeneous, and each individual disorder is represented by one or at most a few individuals. Thirdly, this chart review study is retrospective and “opportunistic”; patients were not systematically screened for cardiac manifestations, and the cardiac studies did not follow a strict protocol but were done on a case-by-case basis. Fourthly, not all IMDs were represented in our cohort; for instance, Fabry disease was missing, and certain diseases, such as long-chain fatty acid oxidation disorders, were underrepresented, which significantly biases the interpretation of the results. This highlights the need for larger cohorts to better represent IMDs with higher cardiac risk. Lastly, it is impossible to unequivocally ascribe long-term complications to the underlying IMD rather than to aging or other co-morbidities. Nevertheless, even with all these limitations in mind, this first exploration with its preliminary indications may serve as a springboard for prospective studies with structured protocols and larger cohorts.

## 5 Conclusion

Improved detection and treatment of IMD over the last decades have resulted in a growing population of adult patients. In this population, we observe complications related to chronic diseases and/or aging that may go beyond the typical features of IMD as originally described in the pediatric literature. This retrospective cohort study suggests that at least 20 % of adults with IMD may have cardiac disease, including both structural heart defects and arrhythmia. Cardiac involvement in IMDs seem not to be rare and may be worthy of prospective studies to determine prevalence and features more precisely. Early detection of cardiac involvement may allow for the prescription of specific cardiac or metabolic treatment and thus to stabilize, improve or even reverse the heart condition. Despite the limitations of this study, our observations support the importance of cardiac screening in IMDs with a significant risk of cardiovascular disease, including GSD III, CblC, PA, MELAS, and KSS. Risk assessment should rely on echocardiography and ECG, as these modalities can identify critical cardiac disease before it becomes symptomatic.

## CRediT authorship contribution statement

**Flutura Sadiku:** Writing – original draft, Resources, Methodology, Formal analysis. **Tobias Rutz:** Writing – review & editing, Validation, Supervision, Software, Methodology, Formal analysis, Data curation, Conceptualization. **Andrea Superti-Furga:** Writing – review & editing, Validation, Supervision. **Pierre Monney:** Writing – review & editing, Validation, Supervision, Methodology, Formal analysis, Conceptualization. **Christel Tran:** Writing – review & editing, Writing – original draft, Visualization, Validation, Supervision, Project administration, Methodology, Formal analysis, Data curation, Conceptualization.

## Consent for publication

All patients provided their written consent to participate in this publication.

## Ethics approval and consent to participate

The research reported in this paper adhered to the Declaration of Helsinki. The protocol was approved by the Swiss Ethics Committees on research involving humans (Approval # 2020–02531). All study participants provided written or oral consent.

## Funding

Open access funding provided by 10.13039/501100006390University of Lausanne.

## Declaration of competing interest

All authors state that they have no competing interests to declare. None of the authors accepted any reimbursements, fees or funds from any organization that may in any way gain or loses financially from the results of this study.

## Data Availability

Data will be made available on request.

## Appendix A Supplementary data

Supplementary material Image 1

## References

[1] Ferreira C.R., Rahman S., Keller M., Zschocke J., Group IA (2021). An international classification of inherited metabolic disorders (ICIMD). J. Inherit. Metab. Dis..

[2] Waters D., Adeloye D., Woolham D., Wastnedge E., Patel S., Rudan I. (2018). Global birth prevalence and mortality from inborn errors of metabolism: a systematic analysis of the evidence. J. Glob. Health.

[3] Ferreira C.R., van Karnebeek C.D.M. (2019). Inborn errors of metabolism. Handb. Clin. Neurol..

[4] Schwarz M., Wendel U. (2005). Inborn errors of metabolism (IEM) in adults. A new challenge to internal medicine. Med. Klin. (Munich).

[5] Saudubray J.M., Sedel F. (2009). Inborn errors of metabolism in adults. Ann. Endocrinol..

[6] Sirrs S., Hollak C., Merkel M., Sechi A., Glamuzina E., Janssen M.C. (2016). The frequencies of different inborn errors of metabolism in adult metabolic centres: report from the SSIEM adult metabolic physicians group. JIMD Rep..

[7] Elliott P., Limongelli G., Hollak C., Lachmann R. (2016). Inherited Metabolic Disease in Adults: A Clinical Guide.

[8] Wicks E.C., Elliott P.M. (2012). Genetics and metabolic cardiomyopathies. Herz.

[9] Conte F., Sam J.E., Lefeber D.J., Passier R. (2023). Metabolic cardiomyopathies and cardiac defects in inherited disorders of carbohydrate metabolism: a systematic review. Int. J. Mol. Sci..

[10] Cuenca-Gomez J.A., Lara-Rojas C.M., Bonilla-Lopez A. (2024). Cardiac manifestations in inherited metabolic diseases. Curr. Probl. Cardiol..

[11] Papadopoulou-Legbelou K., Gogou M., Evangeliou A. (2017). Cardiac manifestations in children with inborn errors of metabolism. Indian Pediatr..

[12] Lloyd D.F., Vara R., Mathur S. (2017). Cardiac manifestations of inherited metabolic disease in children. Pediatr. Int..

[13] Sabate Rotes A., Del Toro Riera M., Albert Brotons D.C., Arranz Amo J.A., Carrascosa Lezcano A., Girona Comas J. (2011). Cardiomyopathy and inborn errors of metabolism in children. Study of 12 cases. Med. Clin. (Barc.).

[14] Evangeliou A., Papadopoulou-Legbelou K., Daphnis E., Ganotakis E., Vavouranakis I., Michailidou H. (2007). Cardiac manifestations of inborn errors of metabolism. Minerva Pediatr..

[15] Cox G.F. (2007). Diagnostic approaches to pediatric cardiomyopathy of metabolic genetic etiologies and their relation to therapy. Prog. Pediatr. Cardiol..

[16] Wang S.M., Hou J.W., Lin J.L. (2006). A retrospective epidemiological and etiological study of metabolic disorders in children with cardiomyopathies. Acta Paediatr. Taiwan..

[17] Brailova M., Clerfond G., Tresorier R., Minet-Quinard R., Durif J., Massoullie G. (2020). Inherited metabolic diseases and cardiac pathology in adults: diagnosis and prevalence in a CardioMetabo study. J. Clin. Med..

[18] Saudubray J.M., Mochel F., Lamari F., Garcia-Cazorla A. (2019). Proposal for a simplified classification of IMD based on a pathophysiological approach: a practical guide for clinicians. J. Inherit. Metab. Dis..

[19] Lang R.M., Badano L.P., Mor-Avi V., Afilalo J., Armstrong A., Ernande L. (2015). Recommendations for cardiac chamber quantification by echocardiography in adults: an update from the American Society of Echocardiography and the European Association of Cardiovascular Imaging. Eur. Heart J. Cardiovasc. Imaging.

[20] Authors/Task Force M, Elliott P.M., Anastasakis A., Borger M.A., Borggrefe M., Cecchi F. (2014). 2014 ESC guidelines on diagnosis and management of hypertrophic cardiomyopathy: the Task Force for the diagnosis and Management of Hypertrophic Cardiomyopathy of the European Society of Cardiology (ESC). Eur. Heart J..

[21] Jenni R., Oechslin E., Schneider J., Attenhofer Jost C., Kaufmann P.A. (2001). Echocardiographic and pathoanatomical characteristics of isolated left ventricular non-compaction: a step towards classification as a distinct cardiomyopathy. Heart.

[22] Petersen S.E., Selvanayagam J.B., Wiesmann F., Robson M.D., Francis J.M., Anderson R.H. (2005). Left ventricular non-compaction: insights from cardiovascular magnetic resonance imaging. J. Am. Coll. Cardiol..

[23] Devereux R.B., de Simone G., Arnett D.K., Best L.G., Boerwinkle E., Howard B.V. (2012). Normal limits in relation to age, body size and gender of two-dimensional echocardiographic aortic root dimensions in persons >/=15 years of age. Am. J. Cardiol..

[24] Humbert M., Kovacs G., Hoeper M.M., Badagliacca R., Berger R.M.F., Brida M. (2022). ESC/ERS guidelines for the diagnosis and treatment of pulmonary hypertension. Eur. Respir. J..

[25] Barbey F., Brakch N., Linhart A., Rosenblatt-Velin N., Jeanrenaud X., Qanadli S. (2006). Cardiac and vascular hypertrophy in Fabry disease: evidence for a new mechanism independent of blood pressure and glycosphingolipid deposition. Arterioscler. Thromb. Vasc. Biol..

[26] Herkert J.C., Verhagen J.M.A., Yotti R., Haghighi A., Phelan D.G., James P.A. (2020). Expanding the clinical and genetic spectrum of ALPK3 variants: phenotypes identified in pediatric cardiomyopathy patients and adults with heterozygous variants. Am. Heart J..

[27] Braunlin E.A., Harmatz P.R., Scarpa M., Furlanetto B., Kampmann C., Loehr J.P. (2011). Cardiac disease in patients with mucopolysaccharidosis: presentation, diagnosis and management. J. Inherit. Metab. Dis..

[28] Rigante D., Segni G. (2002). Cardiac structural involvement in mucopolysaccharidoses. Cardiology.

[29] Fesslova V., Corti P., Sersale G., Rovelli A., Russo P., Mannarino S. (2009). The natural course and the impact of therapies of cardiac involvement in the mucopolysaccharidoses. Cardiol. Young.

[30] Tanpaiboon P., Sloan J.L., Callahan P.F., McAreavey D., Hart P.S., Lichter-Konecki U. (2013). Noncompaction of the ventricular myocardium and hydrops fetalis in cobalamin C disease. JIMD Rep..

[31] Anan R., Nakagawa M., Miyata M., Higuchi I., Nakao S., Suehara M. (1995). Cardiac involvement in mitochondrial diseases. A study on 17 patients with documented mitochondrial DNA defects. Circulation.

[32] Meyers D.E., Basha H.I., Koenig M.K. (2013). Mitochondrial cardiomyopathy: pathophysiology, diagnosis, and management. Tex. Heart Inst. J..

[33] Limongelli G., Masarone D., D’Alessandro R., Elliott P.M. (2012). Mitochondrial diseases and the heart: an overview of molecular basis, diagnosis, treatment and clinical course. Futur. Cardiol..

[34] Khambatta S., Nguyen D.L., Beckman T.J., Wittich C.M. (2014). Kearns-Sayre syndrome: a case series of 35 adults and children. Int. J. Gen. Med..

[35] van Beynum I., Morava E., Taher M., Rodenburg R.J., Karteszi J., Toth K. (2012). Cardiac arrest in Kearns-Sayre syndrome. JIMD Rep..

[36] Imamura T., Sumitomo N., Muraji S., Mori H., Osada Y., Oyanagi T. (2019). The necessity of implantable cardioverter defibrillators in patients with Kearns-Sayre syndrome - systematic review of the articles. Int. J. Cardiol..

[37] Finsterer J. (2019). Cardiac disease in Kearns-Sayre syndrome requires comprehensive management. Cardiol. Young.

[38] Cassiman D., Packman S., Bembi B., Turkia H.B., Al-Sayed M., Schiff M. (2016). Cause of death in patients with chronic visceral and chronic neurovisceral acid sphingomyelinase deficiency (Niemann-Pick disease type B and B variant): literature review and report of new cases. Mol. Genet. Metab..

[39] Lidove O., Belmatoug N., Froissart R., Lavigne C., Durieu I., Mazodier K. (2017). Acid sphingomyelinase deficiency (Niemann-pick disease type B) in adulthood: A retrospective multicentric study of 28 adult cases. Rev. Med. Interne.

[40] McGovern M.M., Aron A., Brodie S.E., Desnick R.J., Wasserstein M.P. (2006). Natural history of Type A Niemann-Pick disease: possible endpoints for therapeutic trials. Neurology.

[41] McGovern M.M., Lippa N., Bagiella E., Schuchman E.H., Desnick R.J., Wasserstein M.P. (2013). Morbidity and mortality in type B Niemann-Pick disease. Genet. Med..

[42] Ferrans V.J., Rodriguez E.R., McAllister H.A. (1985). Granulomatous inflammation of the heart. Heart Vessels Suppl..

[43] Kalil M.A.B., Donis K.C., Poswar F.O., Dos Santos B.B., Santos A.B.S., Schwartz I.V.D. (2020). Cardiovascular findings in classic homocystinuria. Mol. Genet. Metab. Rep..

[44] Evangelisti L., Lucarini L., Attanasio M., Porciani M.C., Romano E., Prisco D. (2009). Vascular and connective tissue features in 5 Italian patients with homocystinuria. Int. J. Cardiol..

[45] Perla-Kajan J., Utyro O., Rusek M., Malinowska A., Sitkiewicz E., Jakubowski H. (2016). N-Homocysteinylation impairs collagen cross-linking in cystathionine -synthase-deficient mice: a novel mechanism of connective tissue abnormalities. FASEB J..

[46] Lorenzini M., Guha N., Davison J.E., Pitcher A., Pandya B., Kemp H. (2018). Isolated aortic root dilation in homocystinuria. J. Inherit. Metab. Dis..

[47] Austin S.L., Proia A.D., Spencer-Manzon M.J., Butany J., Wechsler S.B., Kishnani P.S. (2012). Cardiac pathology in glycogen storage disease type III. JIMD Rep..

[48] Berling E., Laforet P., Wahbi K., Labrune P., Petit F., Ronzitti G. (2021). Narrative review of glycogen storage disorder type III with a focus on neuromuscular, cardiac and therapeutic aspects. J. Inherit. Metab. Dis..

[49] Kishnani P.S., Austin S.L., Abdenur J.E., Arn P., Bali D.S., Boney A. (2014). Diagnosis and management of glycogen storage disease type I: a practice guideline of the American College of Medical Genetics and Genomics. Genet. Med..

[50] Kollberg G., Tulinius M., Gilljam T., Ostman-Smith I., Forsander G., Jotorp P. (2007). Cardiomyopathy and exercise intolerance in muscle glycogen storage disease 0. N. Engl. J. Med..

[51] De Bie I., Nizard S.D., Mitchell G.A. (2009). Fetal dilated cardiomyopathy: an unsuspected presentation of methylmalonic aciduria and hyperhomocystinuria, cblC type. Prenat. Diagn..

[52] Profitlich L.E., Kirmse B., Wasserstein M.P., Diaz G.A., Srivastava S. (2009). High prevalence of structural heart disease in children with cblC-type methylmalonic aciduria and homocystinuria. Mol. Genet. Metab..

[53] Romano S., Valayannopoulos V., Touati G., Jais J.P., Rabier D., de Keyzer Y. (2010). Cardiomyopathies in propionic aciduria are reversible after liver transplantation. J. Pediatr..

[54] Riemersma M., Hazebroek M.R., Helderman-van den Enden A., Salomons G.S., Ferdinandusse S., Brouwers M. (2017). Propionic acidemia as a cause of adult-onset dilated cardiomyopathy. Eur. J. Hum. Genet..

[55] McMurray J.J., Adamopoulos S., Anker S.D., Auricchio A., Bohm M., Dickstein K. (2012). ESC guidelines for the diagnosis and treatment of acute and chronic heart failure 2012: the Task Force for the diagnosis and treatment of acute and chronic heart failure 2012 of the European Society of Cardiology. Developed in collaboration with the heart failure association (HFA) of the ESC. Eur. Heart J..

[56] Braunlin E.A., Berry J.M., Whitley C.B. (2006). Cardiac findings after enzyme replacement therapy for mucopolysaccharidosis type I. Am. J. Cardiol..

[57] Dow D.R., Harper W.F. (1932). The vascularity of the valves of the human heart. J. Anat..

[58] Lampe C., Bosserhoff A.K., Burton B.K., Giugliani R., de Souza C.F., Bittar C. (2014). Long-term experience with enzyme replacement therapy (ERT) in MPS II patients with a severe phenotype: an international case series. J. Inherit. Metab. Dis..

